# Feasibility and acceptability of a bar-based sexual risk reduction intervention for bar patrons in Tshwane, South Africa

**DOI:** 10.1080/17290376.2014.890123

**Published:** 2014-04-22

**Authors:** Neo K. Morojele, Naledi Kitleli, Kgalabi Ngako, Connie T. Kekwaletswe, Sebenzile Nkosi, Katherine Fritz, Charles D.H. Parry

**Affiliations:** ^a^ PhD Psychology, is a Chief Specialist Scientist at the Alcohol and Drug Abuse Research Unit, Medical Research Council, Pretoria, South Africa; ^b^ Honorary Associate Professor at the School of Public Health, University of the Witwatersrand, Johannesburg, South Africa; ^c^ BA (Hons) Psychology, is a Scientist at the Alcohol and Drug Abuse Research Unit, Medical Research Council, Pretoria, South Africa; ^d^ M Cur, is a Chief Research Technologist at the Alcohol and Drug Abuse Research Unit, Medical Research Council, Pretoria, South Africa; ^e^ PhD Clinical Health Psychology, is a Senior Scientist at the Alcohol and Drug Abuse Research Unit, Medical Research Council, Pretoria, South Africa; ^f^ MA Psychology, is a Scientist at the Alcohol and Drug Abuse Research Unit, Medical Research Council, Pretoria, South Africa; ^g^ PhD Social and Cultural Anthropology, is the Director of Global Health at the International Center for Research on Women, Washington, DC, USA; ^h^ PhD Community Psychology, is the Unit Director at Alcohol and Drug Abuse Research Unit, Medical Research Council, Cape Town, South Africa; ^i^ Extraordinary Professor at the Department of Psychiatry, University of Stellenbosch, Tygerberg, South Africa

**Keywords:** HIV prevention, bar-based intervention, server intervention, peer intervention, brief intervention, motivational interviewing, prévention VIH, intervention dans des bars, intervention des serveurs, intervention des pairs, brève intervention, entretien de motivation

## Abstract

Alcohol consumption is a recognised risk factor for HIV infection. Alcohol serving establishments have been identified as appropriate venues in which to deliver HIV prevention interventions. This paper describes experiences and lessons learnt from implementing a combined HIV prevention intervention in bar settings in one city- and one township-based bar in Tshwane, South Africa. The intervention consisted of peer-led and brief intervention counselling sub-components. Thirty-nine bar patrons were recruited and trained, and delivered HIV and alcohol risk reduction activities to their peers as peer interventionists. At the same time, nine counsellors received training and visited the bars weekly to provide brief motivational interviewing counselling, advice, and referrals to the patrons of the bars. A responsible server sub-component that had also been planned was not delivered as it was not feasible to train the staff in the two participating bars. Over the eight-month period the counsellors were approached by and provided advice and counselling for alcohol and sexual risk-related problems to 111 bar patrons. The peer interventionists reported 1323 risk reduction interactions with their fellow bar patrons during the same period. The intervention was overall well received and suggests that bar patrons and servers can accept a myriad of intervention activities to reduce sexual risk behaviour within their drinking settings. However, HIV- and AIDS-related stigma hindered participation in certain intervention activities in some instances. The buy-in that we received from the relevant stakeholders (i.e. bar owners/managers and patrons, and the community at large) was an important contributor to the feasibility and acceptability of the intervention.

## Résumé

La consommation d'alcool est un facteur à risque reconnu de transmission de l'infection du VIH. Les établissements servant de l'alcool ont été identifiés comme des lieux appropriés pour effectuer des interventions de prévention contre le VIH. Cet article décrit les expériences et les leçons tirées de l'implémentation d'une intervention combinée de prévention contre le VIH dans un bar de la ville et un bar du township de Tshwane en Afrique du Sud. L'intervention consistait en de brefs sous-composants de conseils donnés par des pairs. Trente neuf clients de bars ont été recrutés et formés et ont reçus des activités liées à la réduction de l'alcool et du risque de VIH pour les transmettre à leurs pairs en tant qu'interventionnistes. En même temps, neuf conseillers ont été formés et sont allés dans ces bars toutes les semaines pour donner de brefs conseils, avis et recommandations concernant les entretiens de motivation (EM) aux clients de bars. Un sous-composant de serveurs responsables qui avait également été programmé n'a pas été transmis étant donné qu'il n’était pas possible de former le personnel de deux bars participants. Au cours des huit mois, les conseillers ont été abordés et ont donné des conseils et des avis concernant les problèmes liés aux risques sexuels et à la consommation d'alcool à 111 clients de bars. Les interventionnistes ont signalé 1 323 interventions de réduction des risques auprès de leurs clients de bars durant la même période. L'intervention a globalement été bien reçue et suggère que les clients de bars et serveurs peuvent accepter bon nombre d'activités d'intervention afin de réduire les comportements sexuels à risque dans les établissements de consommation d'alcool. Cependant, dans certains cas, la stigmatisation liée au VIH et au SIDA a entravé la participation de certaines activités d'interventions. Le bénéfice que nous avons reçu des intervenants concernés (à savoir propriétaires de bars et clients, et la communauté dans son ensemble) était un facteur important de la faisabilité et de l'acceptation de l'intervention.

## Introduction

South Africa is at the epicentre of the HIV epidemic, with among the highest HIV prevalence rates in the world (UNAIDS & WHO 2009). South Africa is faced with a double burden of heavy alcohol consumption and HIV infection. Recent reports reveal that South Africans also have among the highest rates of per capita alcohol consumption (Roerecke, Obot, Petra & Rehm 2008), and drinking in South Africa is characterised by episodic binge drinking; a style of drinking most strongly associated with problem outcomes (WHO 2004). A recent meta-analysis of studies conducted in sub-Saharan countries, including South Africa, revealed that alcohol consumption, and particularly ‘problem drinking’ is associated with HIV infection (Fisher, Bang & Kapiga 2007). Other studies have shown increased involvement in sexual risk behaviour among alcohol users (Kalichman, Simbayi, Kaufman, Cain & Jooste 2007; Neuman, Schneider, Nanau, Parry & Chersich 2012; Shuper, Neuman, Kanteres, Baliunus, Joharchi & Rehm 2010; Woolf-King & Maisto 2011). Previous research has suggested that the relationships between alcohol consumption and sexual risk behaviour are complex and are a function of the patterns of alcohol consumption, characteristics of consumers, and contexts/settings in which alcohol consumption takes place (Kalichman *et al*. 2007; Morojele, Kachieng'a, Mokoko, Nkoko, Parry, Nkowane, *et al*. 2006; Morojele, Kachieng'a, Nkoko, Moshia, Mokoko, Parry, *et al*. 2004). Bars are one setting in which alcohol is commonly associated with the initiation of (and often the engagement in) sexual encounters, many of which are risky. Such settings have been identified as ideal for implementation of sexual risk reduction interventions, since they are places at which heavy alcohol users are found in large numbers, risky sexual behaviours occur, and places which individuals voluntarily frequent and comprise a captive audience (Lewis, Garnett, Mhlanga, Nyamupuka, Donelly & Gregson 2005; Morojele *et al*. 2006; Sandøy, Siziya & Fylkesnes 2008; Weir, Pailman, Mahlalela, Coetzee, Meidany & Boerman 2003). On the assumption that alcohol consumption is likely to be causally related to sexual risk behaviour, reductions in alcohol consumption can be expected to lead to reductions in sexual risk behaviour, and consequently, HIV infection (Parry, Rehm & Morojele 2010). Consequently, a bar-based HIV intervention which would include heavy drinkers who are at higher risk for HIV infection should target both sexual risk behaviour and alcohol use.

This paper describes our experiences in implementing an HIV prevention programme designed to reduce alcohol use and sexual risk behaviours associated with HIV infection among bar patrons in South Africa. The intervention was named the ‘Vuwani Programme’. Vuwani is a TshiVenda term which means ‘wake up’. The Vuwani intervention was developed on the basis of our review of the literature on health behaviour change interventions that have been conducted in bar settings (Fritz & Lawrence 2009), and formative research in community settings (Morojele, Fritz, Parry, Manda & Nel 2007). From the literature review we identified three main types of alcohol and/or sexual risk reduction intervention approaches that have been conducted in bar settings: (1) server interventions to reduce harmful alcohol use, (2) peer-led interventions to reduce sexual risk behaviour, and (3) brief interventions to reduce harmful alcohol use.

*Server training interventions* aim to train and equip bar owners and managers, as well as staff who sell and serve alcohol (servers), with skills and knowledge to promote responsible drinking and avert the harms associated with drinking to intoxication among bar patrons. Server interventions have shown promise, but so far have been inconclusive, in that intervention studies are yet to establish a link between server behaviour and behaviour change among bar patrons (Ker & Chinnock 2006). For example, Peltzer, Ramlagan and Gliksman (2006) conducted a server intervention in bars in townships in Cape Town (South Africa). The intervention had positive effects on server knowledge and attitudes, as well as some indications of positive changes in server behaviours, but it did not have positive effects on bar patrons' levels of intoxication.

*Peer-led interventions* in bars have involved the recruitment and training of individuals – often referred to as ‘popular opinion leaders’ (POLs; Kelly, Lawrence, Diaz, Stevenson, Hauth, Brasfield, *et al*. 1991) – to be agents of change within their social networks. The POL model has been used in several trials in gay bars in the USA with some success (Kegeles, Hayes & Coates 1996; Kelly, Murphy, Sikkema, McAuliffe, Roffman, Solomon, *et al*. 1997). However, this intervention model proved ineffective in gay bars in Scotland (Flowers, Hart, Williamson, Frankis & Der 2002; Williamson, Hart, Flowers, Frankis & Der 2001). Other researchers have used a modified version of this model, referred to as the Community POL (C-POL) intervention. For example, a modified version of the POL model was tested in a randomised controlled trial in beerhalls in Harare (Zimbabwe), results of which indicated minimal effectiveness (Fritz, McFarland, Wyrod, Chasakara, Makumbe, Chirowodza, *et al*. 2011). Also of relevance is a recent National Institutes of Health-funded worldwide trial of the C-POL intervention conducted in Peru, India, Zimbabwe, Russia, and China (NIMH Collaborative HIV/STD Prevention Trial Group 2010). The study found reductions between baseline and a 24-month follow-up point in risk behaviour and sexually transmitted infections (STIs) incidence among participants in both the C-POL venues and the AIDS education (comparison) venues. However, the reductions were not significantly different. The authors suggested that the change (reduction) in the comparison group could be due to their exposure to ‘a substantial and sustained AIDS education intervention’ (212) and to external prevention initiatives.

*Brief interventions* for addressing alcohol consumption provide screening for hazardous or harmful alcohol consumption, as well as brief counselling, information, advice, and referral (Babor & Higgins-Biddle 2001). Brief interventions can vary from one very short session to several consecutive sessions, and can be implemented in both healthcare and community settings (Reilly, Van Beurden, Mitchell, Dight, Scott & Beard 1998). Bar-based brief interventions can be described as ‘opportunistic brief interventions’. We located only one example of a bar-based brief intervention programme globally (Reilly *et al*. 1998; Van Beurden, Reilly, Dight, Mitchell & Beard 2000). Van Beurden *et al*. (2000) conducted a brief intervention to reduce risky alcohol consumption in bars in Australia and showed reductions in alcohol consumption after 12 months. Another study that is of relevance, since it included bar patrons, evaluated a brief intervention to reduce levels of sexual risk behaviour among patrons of bars in townships in Cape Town, South Africa (Kalichman, Simbayi, Vermaak, Cain, Smith, Mthembu, *et al*. 2008). The intervention was not venue-based per se, as the counselling was provided off-site. At a three-month follow-up point the authors found an increase in condom use and a decrease in alcohol use before sex, although these changes were not sustained in the longer term.

On the basis of our literature review, we concluded that none of the above three approaches alone would be sufficient to reduce alcohol and sexual risk behaviours among patrons of drinking venues. Consequently, we developed a hybrid, multi-level, intervention (the Vuwani Programme) that has individual, structural, and social influence elements (Fritz, Morojele & Kalichman 2010; Kalichman 2010), all of which were designed to operate interdependently. The sub-components include (1) training of servers to encourage responsible drinking and sexual risk reduction; (2) identification and training of suitable bar patrons to serve as peer interventionists or ‘change agents’; (3) deploying counsellors to the drinking venues to conduct screening and provide education, advice, and counselling, as well as referrals to formal counselling and treatment services; (4) assisting bar owners/managers to establish physical conditions that foster responsible drinking and risk reduction; (5) disseminating prevention messages and information via posters, leaflets, and other media; and (6) making affordable and free condoms consistently available at drinking venues. This paper describes the feasibility and acceptability of implementation of the Vuwani intervention in two drinking venues, one in a township and one in a city site, in Tshwane Municipality, Gauteng province, South Africa. Outcomes of the intervention were not assessed due to funding challenges. For the researchers' convenience, the study was conducted in Gauteng province, which had HIV prevalence rates in 2008 of 10.1%, 14.4%, and 15.2% for individuals aged 15–24 years, 25+ years, and 15–49 years, respectively (Shisana, Rehle, Simbayi, Zuma, Jooste, Pillay-van-Wyk, *et al*. 2009).

## Methods

The intervention was implemented in one city- and one township-based licensed drinking establishment over eight months (December 2009 to July 2010). The two venues were selected after a number of processes. We initially received permission to conduct participant observations in the bars from the owners/managers of 6 of the 12 licensed venues in an area of the city site, and 9 of the 10 licensed venues in the selected township site. Bar owners/managers who did not grant permission were mainly concerned about the intervention's effects on their profit. On the basis of the participant observations, we identified and selected the intervention venues, which had to meet the following criteria: (a) at least 30 patrons on a normal week day (not month end); (b) at least one-quarter of the patrons comprising females; and (c) a relatively stable clientele over time. These criteria were selected to ensure that the intervention would be conducted in venues with relatively large numbers of clients, and in which both men and women converged, given the intervention's focus on risk modification of heterosexual transmission of HIV. The project was conducted in partnership with a Community Advisory Board (CAB), comprising members of the two study communities.

### Server intervention

The server intervention sub-component was modelled on standard responsible alcohol service programmes and was designed to train all staff and managers to serve and sell alcohol responsibly, discourage intoxication, and comply with the alcohol legislation of South Africa. It also aimed to train staff to promote condom use and HIV prevention activities, and promote and support the peer and counsellor sub-components of the overall intervention.

### Peer intervention

The peer intervention sub-component was designed to be carried out by regular and popular patrons of the selected bars or POLs. We referred to the POLs as Sisonke Mentors; a name that was selected by our CAB. *Sisonke* is an isiZulu word meaning ‘we are together’, and ‘Mentor’ captures the essence of the POLs' key roles. This intervention sub-component, with its focus on alcohol and HIV risk reduction, sought to adopt aspects of the nine core elements of Kelly's POL model which are shown in Table 1 (Kelly 2004).

**Table 1.** Core elements of the POL model.

1. Intervention directed to identifiable target group in well-defined community venues where population size can be estimated
2. Use of ethnographic techniques to identify POLs
3. 15% of target population size of venues are trained as POLs
4. Programme teaches POLs skills for initiating HIV risk reduction messages during everyday conversations
5. Training teaches POLs characteristics of effective behaviour change communication messages (targeting risk-related attitudes, norms, intentions, and self-efficacy); POLs taught to endorse benefits of safer behaviour and recommend practical steps needed to implement change
6. Groups of POLs meet weekly to help them refine their skills and gain confidence in delivering effective intervention messages. Groups should be sufficiently small to enable POLS to practice, shape their communication skills, and create comfort in delivering conversational messages
7. POLs set goals to engage in risk reduction conversations between weekly sessions
8. POLs' conversational outcomes are reviewed, discussed, and reinforced at subsequent training sessions
9. Logos, symbols, and other devices used as conversation starters between POLs and others

Source: Kelly (2004).

We recruited the Sisonke Mentors via a process of nomination among the patrons and managers of the bars. Eligibility criteria for the Sisonke Mentors included being (a) at least 18 years of age; (b) a regular visitor of the bar (at least twice per week); (c) willing and able to attend a two-day training workshop; (d) a ‘people's person’; (d) a ‘moderate’ drinker; (e) pro-HIV prevention; and (d) willing to volunteer time as a Sisonke Mentor for six months. Recipients of the most nominations were chosen, and those who were willing to serve as Sisonke Mentors signed consent forms and then underwent training.

Nine patrons at the township bar and 30 at the city bar were nominated and volunteered to become Sisonke Mentors. In the township they comprised 4 females and 5 males, aged between 23 and 37 years, and in the city they consisted of 16 females and 14 males aged between 20 and 40 years.

Training occurred in nine waves, as new groups of patrons were enrolled over time. Each group of trainees attended two, one-day training sessions, separated by one week. Training was both didactic and interactive and incorporated core elements of the POL model. All training sessions were scheduled for Sundays between 09:00 and 15:30, which was the most suitable time for the nominees. Topics of Day 1 sessions included (1) Introduction and overview; (2) Introduction to roles and responsibilities (to address POL Element 4); (3) Alcohol use and abuse (Element 5); (4) Links between alcohol use and sexual risk behaviour (to address Elements 4 and 5); (5) Alcohol legislation, to reinforce the Sisonke Mentors' alcohol reduction messages/efforts; (6) HIV biology (Element 5); and (7) HIV transmission (Element 5). On Day 2 the training focused on (1) Reviewing Day 1's content; (2) Roles and responsibilities (Element 4); and (3) Skills building (Element 4). Training was conducted primarily by the study's personnel, but also involved personnel from a social change communication non-governmental organisation to train on HIV/AIDS biology and transmission, and from the Gauteng Department of Economic Development to train on the legislation regarding the sale of alcohol. At the end of training the Sisonke Mentors were given an attendance certificate, a blue project T-shirt (Element 9), reading material, a bag, a male condom demonstrator model, and male and female condoms, and they completed a training evaluation form.

During the intervention period the Sisonke Mentors attended fortnightly support/debriefing sessions at the bars on Sunday afternoons. In keeping with POL Element 6, these meetings were held to promote ownership of the Vuwani project among the Sisonke Mentors; provide ongoing training and support; and serve as a forum for them to share the challenges they faced in intervention delivery. They also served to monitor whether the Sisonke Mentors delivered the intervention as intended, by eliciting the Sisonke Mentors' feedback on their activities for the previous two weeks and providing skills building and problem-solving. There was no direct observation of the Sisonke Mentors while interacting with bar patrons since this intervention component, which was conducted during the Sisonke Mentors' normal venue visits, was meant to be as naturalistic as possible, and weaved into the normal conversations they would otherwise have with their peers. During the support sessions the Sisonke Mentors also completed and handed in a structured self-reporting form on which they indicated the number of male and female bar patrons with whom they had interacted during the previous fortnight, as well as the nature of the interactions. After each session they received a voucher valued at R100 (approximately US$14) for shops that sell toiletries, homeware and medications, and that were selected because they do not sell alcoholic beverages. At the end of the project the Sisonke Mentors from both sites attended a combined feedback session in order to share their experiences on the project.

### Counsellor intervention

We recruited and trained nine counsellors with experience in substance abuse and/or HIV/AIDS all of whom remained active throughout the project period. In the township they comprised four females and one male, aged between 28 and 35 years, and in the city they consisted of two females and two males aged between 30 and 41 years.

The counsellors received training in brief intervention with a focus on motivational interviewing (MI; Miller & Rollnick 1991). Training was conducted by a registered MI trainer and took place over two days in November 2009. Training involved the following sessions: (a) Introduction; (b) Review of the basics of the helping relationship; (c) Introduction to principles of brief interventions; (d) Introduction to MI as a form of brief intervention; (e) MI: goals, spirit, principles, approach and skills; (f) basic principles of substance abuse; (g) problem drinking and how to screen for it; (h) HIV risk behaviour and how to screen for it; (i) strategies for effective communication with bar patrons; (j) risk reduction plans; (k) principles of referral (matching); (l) role plays; and (m) discussions and evaluation. A half-day booster training took place in May 2010.

Within each bar, the counsellors delivered the intervention in pairs on Saturdays or Sundays for six hours per visit. At the venues they would sit at a table, on which they displayed health promotional materials, and male and female condoms, and wait to be approached by bar patrons. Bar patrons who approached the counsellors were screened for alcohol problems by using the four-item CAGE (Mayfield, McLeod & Hall 1974) or the five-item TWEAK (Russell, Martier, Sokol, Mudler, Bottoms, Jacobson, *et al*. 1994) instruments. The TWEAK was designed specifically for pregnant women (Russell *et al*. 1994) and was used in this study as it is appropriate for assessing alcohol problems among women in general. Screening was followed by provision of a brief, 5–10 minute, MI counselling session, if appropriate. The counsellors referred patrons to outside services where necessary, and each approaching patron was given a ‘resource list’, with contact numbers of organisations concerned with alcohol, HIV/AIDS, and other related issues.

The counsellors were required to document details, on a semi-structured self-reporting form, about each interaction with a bar patron, and each visit to a bar. Debriefing took place after each venue visit. In addition, joint support sessions with counsellors from both sites were conducted after every six weeks on Saturday afternoons in part, as a quality control measure, and to provide skills building and problem-solving for challenges encountered during their interactions with bar patrons. There was no direct observation of the counsellors while intervening with bar patrons, since this would have undermined patron–counsellor confidentiality and may have made the patrons feel uncomfortable about being open with the counsellors. Instead, the supervisors who accompanied the counsellors simply observed the general nature and context of the interactions (e.g. body language, duration).

The research was approved by the Ethics Committee of the Faculty of Health Sciences of the University of Pretoria (Protocol Number 3/2008).

## Results

### Server intervention

The serving staff of both bars did not undergo training as planned. However, they displayed great interest in the project and the managers permitted us to conduct all other intervention activities in their establishments. The township bar's two staff members who served and sold alcohol were unable to find time to attend training due to their daily work commitments. The city-based bar had numerous staff (approximately 30) comprising the manager, servers, cleaners and door/security staff. At the project's onset, the manager did not wish his staff to receive training, fearing negative financial effects on his business. Towards the end of the intervention period, however, he became increasingly interested in the project's outcomes and requested training for his staff, but this was not possible due to limited time.

### ‘Sisonke Mentor’ (peer) intervention

Recruitment of the Sisonke Mentors was time-consuming in requiring numerous return visits to the bars to explain the nomination process to the bar patrons and ask the bar managers to suggest names of potentially appropriate candidates. We finally recruited 39 bar patrons in the 2 bars. The township bar had approximately 20 regular bar patrons, and the 9 individuals who were trained as Sisonke Mentors made up 45% of the regular bar patrons; this percentage exceeds the stipulated 15% of the total number of regular patrons (Kelly 2004). The city-based bar had a maximum capacity of 500 people. It is not clear how many of the people who visited the bar over the intervention period were regular patrons, but the number of patrons who were eventually trained comprised approximately 6% (30/500) of the target population, and hence did not meet the 15% requirement (Kelly 2004).

Most Sisonke Mentors evaluated their training very positively. They reported that the sessions on HIV/AIDS and STIs, and Sisonke Mentors' rights, roles, and responsibilities were particularly useful. After training most participants (91.4%) reported knowing what was expected of them (‘a lot’) and feeling excited (94.1%) and happy (100%). Also, levels of confusion (15.4%) and fear (11.5%) were low, although half of the trainees reported some nervousness (50%). Nonetheless, the Sisonke Mentors' anxiety levels dissipated over time as witnessed through their participation during the support sessions and their interactions with their fellow bar patrons.

The Sisonke Mentors visited the bars on average 4.39 (SD = 2.5) times per fortnight during the intervention period. They reported on the number and nature of interactions they had during the two-week period. As shown in Table 2, the Sisonke Mentors had interactions with a maximum of 746 males and 577 females throughout the entire 8-month intervention period. However, these figures may be over-estimates since they are based on summing the numbers of people reportedly reached each fortnight over the entire project period. It was not possible to avoid double counting as the Sisonke Mentors were required to simply sum the number of people they interacted with on each day during each two-week period. According to their reports, shown in Table 2, the Sisonke Mentors were most likely to encourage or facilitate condom use among their male and female peers, but least likely to escort any peers home, advise them to seek the counsellors' advice, or advise the peers to seek STI treatment.

**Table 2.** Male and female bar patrons with whom the Sisonke Mentors (peers) interacted on various prevention activities over an eight-month period.

Alcohol and HIV prevention activity	Males	Females
Advised someone at the bar/tavern to use condoms	746	577
Assisted someone at the bar/tavern to get condoms (either gave one or helped buy one)	695	424
Advised someone at the bar/tavern to limit their alcohol use	691	494
Advised someone at the bar/tavern to be faithful to their wife/husband or regular partner	566	464
Discussed with someone at the bar/tavern HIV and ways to help prevent infection	505	360
Advised someone at the bar/tavern not to have sex with a casual sexual partner	492	378
Encouraged someone at the bar/tavern to get an HIV test	444	345
Advised someone at the bar/tavern to go home from the bar/tavern early	430	370
Gave someone at the bar/tavern informational material about HIV or other STIs	406	339
Discussed with someone at the bar/tavern the option to abstain from sex	387	299
Gave someone at the venue a resource list	339	250
Gave condom demonstration to someone at the bar/tavern	329	204
Distracted or physically disrupted someone at the bar/tavern from hooking up with a casual sexual partner	302	217
Advised someone at the bar/tavern to consult the counsellors	301	191
Advised someone at the bar/tavern to seek STI treatment	233	161
Escorted someone at the bar/tavern home	183	195
Sought information/advice from the counsellors	161	99

All the Sisonke Mentors who received training continued participating in the project throughout the intervention period. Those in the city site in particular had a lot of initiative and innovative ideas on how to enhance their effectiveness. For example, they designed a board game for bars for promoting responsible sexual behaviour and alcohol use, and planned and ran the closing social function for the bar at the end of the intervention period.

The Sisonke Mentors were well accepted by their peers. They were easily identifiable from the blue branded T-shirts that they wore; although sometimes mistaken as HIV positive or in the bar to conduct HIV tests. Communicating with their peers was sometimes hampered by (a) language barriers among foreign Sisonke Mentors who could not speak local South African languages; (b) difficulties in engaging non-regular bar patrons; (c) high noise levels in the venues; and (d) their approaches being misinterpreted as romantic or sexual advances.

The support sessions were generally well attended, but often started late due to the late arrival of Sisonke Mentors, particularly in the township site. Some Sisonke Mentors felt that completing the self-reporting form was tedious and time-consuming.

The Sisonke Mentors reported feeling that they were impacting positively on people, and even ‘saving lives’ by conducting their activities. They reported that they benefitted personally from the project, and (a) gained a sense of value by sharing information with members of the community; (b) acquired valuable knowledge and skills about condom use, HIV, and AIDS; and (c) in a few cases, reduced their own alcohol consumption.

### Counsellors

The counsellors reported that the training had been effective, informative, and had equipped them with skills to encourage behaviour change during their visits to the bars. They reported that the mid-project booster training was extremely useful for reinforcing their skills and for trouble-shooting the challenges they experienced in the bars.

Uptake of the counsellors' services was slow initially, but gradually increased over time. A total of 111 bar patrons, most of whom were males, approached the counsellors during the eight-month period (Table 3). Most patrons (92.3%) approached the counsellors for their own rather than others' personal problems. They mostly approached the counsellors for assistance/advice regarding HIV/AIDS-related matters (60.7%), followed by substance abuse-related problems (30.8%), and there were many requests for an HIV test. The counsellors gave out 1880 male and 178 female condoms in the township venue and 2264 male and 142 female condoms in the city bar. They conducted 18 and 14 male condom demonstrations in the city and township bars, respectively, and 19 and 11 female condom demonstrations, in the city and township bars, respectively.

**Table 3.** Characteristics of the bar patrons who approached the counsellors over the eight-month period.

	City (*N* = 63)	Township (*N* = 48)	Total (*N* = 111)
	*N* (%)	*N* (%)	*N* (%)
*Gender of patrons*			
Male	45 (71.4)	40 (83.3)	85 (76.6)
Female	18 (28.6)	8 (16.7)	26 (23.4)
*For whom patron sought assistance*^a^			
Self	57 (90.5)	46 (95.8)	103 (92.8)
Other	7 (11.3)	7 (15.2)	14 (13.0)
*Patron's problem*^a^			
HIV-related	35 (59.3)	30 (62.5)	65 (60.7)
Substance abuse	12 (20.3)	21 (43.8)	33 (30.8)
Mental health	2 (3.4)	3 (6.3)	5 (4.7)
Other	12 (20.3)	9 (19.6)	21 (20.0)
*Patrons referred*^a^	49 (77.8)	22 (47.8)	71 (65.1)
Issue for which referral made^a^			
HIV	32 (57.1)	13 (31.0)	45 (45.9)
Substance abuse	13 (23.2)	9 (22.0)	22 (22.7)
Mental health	2 (3.6)	0 (0)	2 (2.1)
Other	6 (10.7)	2 (4.8)	8 (8.2)

^a^Multiple responses endorsed in case of some patrons. Missing data present in some cases.

The counsellors all agreed that brief MI was ideal for use in the bars because it is of very short duration. The non-confrontational approach enables patrons to seek information voluntarily and at their leisure. However, some counsellors would have preferred a more proactive approach as they felt that some bar patrons lacked courage to approach them. Regarding their experiences in applying the four main MI approaches, the counsellors reported having encountered challenges in (a) emphasising to the clients that they have control (49%); (b) asking for permission to screen and give screening feedback (34%); (c) supporting the client (32%); and (d) affirming the client (30%) (Table 4). The township-based counsellors found it more difficult than the city-based counsellors to adopt these MI-type approaches.

**Table 4.** Percentages of clients with whom adoption of various MI counselling approaches was challenging for the counsellors.

	City	Township	Total
Asking for permission	26	47	34
Affirming client	17	50	30
Emphasising client's control	47	53	49
Supporting client	20	50	32

The counsellors felt that the materials and condoms displayed during the site visits were informative and educational. However, they felt that these were not sufficiently attractive or varied, and should also have dealt with a wider range of other health and wellness-related issues (such as illicit drugs, how to quit smoking, and nutrition).

HIV and AIDS stigma and negative perceptions about counselling seemed to prevent some of the bar patrons from going to the counsellors' table to collect materials and/or to converse with or be counselled by the counsellors. This was especially because the counsellors were stationed in full view of other patrons in the bars, but this reluctance reduced over time.

To address the initial slow uptake of the counsellors' services, we increased the duration and frequency of the counsellors' visits, and assigned the same individuals to the same venue consistently. This allowed rapport to develop between the bar patrons and the counsellors, and eventually, we observed more frequent approaches being made.

The intervention was designed such that each sub-component was inter-related. During the early stages of the programme, the Sisonke Mentors rarely interacted with the counsellors but over time, interaction increased, and the counsellors even attended the Sisonke Mentors' support sessions. Approaches to the counsellors increased after this, and some bar patrons informed the counsellors that they had consulted them precisely because they had learned about their role from the Sisonke Mentors.

The counsellors felt that the bar patrons responded positively to their presence overall. The bar setting was considered to be appropriate for accessing people in need of interventions, although bar patrons who visit bars to relax and socialise might be loath to deal with their personal problems each time they visit bars. High noise levels often prevented effective/audible counsellor–patron interactions (especially during football matches), but the counsellors usually managed to improvise and identify conducive counselling spaces in the bars.

## Discussion

This paper responds to the growing call for venue-based HIV risk reduction interventions for alcohol users who are at heightened risk for HIV infection in Africa (e.g. Kalichman 2010). This is one of the first studies to demonstrate the feasibility and acceptability of implementing such an intervention in bars, in a city, and a township site, in South Africa. Two out of three of the main intervention sub-components were implemented successfully (the peer and counsellor sub-components), while the third sub-component was never delivered. In addition, a large number of male and female condoms were dispensed and many condom demonstrations were conducted within the drinking venues.

The bar owners and managers accommodated all of the project's sub-elements except for the ones that involved them personally. They did not attend the server training workshops due to staff shortages, a lack of time and/or interest. Difficulties with the enlisting of bar staff to take part in or comply with server interventions are not uncommon (Ker & Chinnock 2006), although our experiences differed from those of a study in South Africa which showed success in this regard (Peltzer *et al*. 2006). Given HIV stigma, the HIV component of this project, which is usually absent in server training programmes, may have contributed to the bar managers' limited personal involvement in the current project. Although we cannot be certain, it is possible that if the server intervention had been implemented, with its focus on encouraging responsible drinking and discouraging intoxication, we may have seen a greater emphasis on alcohol reduction activities. Further work is needed to determine how to address barriers preventing bar staff's active participation in server training activities as part of bar-based HIV prevention interventions. Possible strategies include offering very short training sessions at the bars themselves and providing incentives to mitigate loss of earnings during training and opportunities for generating income via other means.

The project was successful in recruiting, training, and retaining a large number (39) of bar patrons as peer interventionists (Sisonke Mentors) from both sites for the entire intervention period. In addition, our peer intervention component met most of the nine elements of Kelly's (2004) POL model. The excellent attendance rates at the fortnightly support meetings were attributable to high levels of enthusiasm among the peers, as well as our regular reminders of the meetings, provision of incentives at the end of the meetings, and the fact that most peers were regular visitors at the venues to begin with. The Sisonke Mentors were most active in encouraging condom use and the procurement of condoms for their peers, but involved themselves less in matters requiring greater effort or personal involvement (e.g. escorting people home and advising them to use the counsellors' services). These outcomes suggest that peer interventionists should be provided with ongoing and interactive training and support during which their roles and responsibilities are repeatedly clarified, and skills to interact with peers are constantly reinforced.

The counsellor sub-component was also feasible and acceptable within the bar context. The uptake of the counsellors' services was low initially but increased over time, as the bar patrons became comfortable with the counsellors' presence. By the end of the eight-month intervention period, and despite high noise levels, 111 people had approached the counsellors for advice and to discuss and/or be counselled about HIV and AIDS, alcohol and other drug problems, and other general psycho-social concerns. Of note were the numerous requests for HIV testing, which we did not provide as part of the intervention programme. This suggests that consideration should be given to providing HIV counselling and testing (HCT) in future bar-based intervention projects. We facilitated testing for those who made such requests, by referring them to community resources which render free HCT services.

The success of the counsellor intervention sub-component is attributable to (a) carefully planning and tailoring the frequency and timing of the counsellors' visits to the bar patrons' and staff's comfort levels; (b) stationing the same individual counsellors in the same venues during consecutive weeks, which facilitated the development and maintenance of rapport with bar patrons; (c) utilising diverse health promotional materials in order to decrease discomfort and stigma that would have resulted if the content had been limited to HIV and AIDS topics; (d) using individuals from outside the project communities as counsellors; and (e) the provision of regular debriefing and top-up training to the counsellors for encouragement and support.

Increased use of the counsellors' services may be enhanced by providing a private and quiet space within or near to the bar which is conducive to counselling interactions. In addition, measures are needed to enhance the counsellors' comfort in utilising the main MI techniques (emphasising their clients' control over behaviour change; asking their clients for permission to conduct screening and give screening feedback; and affirming their client). Previous studies have documented similar difficulties among counsellors and interventionists who have prior experience in more directional and non-client-centred counselling approaches. The township-based counsellors reported greater difficulty adopting these approaches than their counterparts in the city. This may be due to a combination of factors, such as (a) milieu differences, as the township-based patrons were older, and likely to be more conservative and unaccustomed to solving personal problems outside of the family context; and (b) counsellor personality and experience differences, as the township-based counsellors had had less experience conducting MI counselling in bar settings on this overall project.

Our project was limited by our failure to use sufficient objective fidelity measures, rather than self-reports. The completion of the self-reporting forms was described as tedious, especially by the Sisonke Mentors. Better methods are needed to more accurately capture information about intervention fidelity.

The insights gained from implementing the Vuwani intervention allow us to offer the following recommendations for implementing combined HIV interventions in bar settings. First, bar patrons and servers can accept a myriad of intervention activities to reduce sexual risk behaviour within their drinking settings. Second, measures should be taken to mitigate HIV- and AIDS-related stigma that may hinder participation in certain intervention activities in bars. Third, it is advisable, where appropriate, to deliver intervention activities in an edutainment-type style that is consistent with people's primary (relaxing, socialising, and entertainment) motivation for going to drinking venues (Kalichman 2010). Fourth, it is crucial that the interdependence of the programme's sub-components is clearly recognised by all participants involved in delivering multi-component intervention programmes. Fifth, ensuring the buy-in of relevant stakeholders (e.g. bar owners/managers and servers, patrons, and the community at large) is crucial for the feasibility of such projects. Finally, visiting the bars/venues regularly and frequently, and for long durations is likely to enhance project buy-in among bar patrons.

## References

[CIT0001] Babor T. F., Higgins-Biddle J. C. (2001). Brief Intervention for Hazardous and Harmful Drinking: A Manual for Use in Primary Care.

[CIT0002] Fisher J. C., Bang H., Kapiga S. H. (2007). The Association between HIV Infection and Alcohol Use: A Systematic Review and Meta-Analysis of African Studies. Sexually Transmitted Diseases.

[CIT0003] Flowers P., Hart G. J., Williamson L. M., Frankis J. S., Der G. J. (2002). Does Bar-Based, Peer-Led Sexual Health Promotion have a Community-Level Effect amongst Gay Men in Scotland?. International Journal of STD & AIDS.

[CIT0004] Fritz K., Lawrence X. (2009).

[CIT0005] Fritz K., Morojele N., Kalichman S. (2010). http://dx.doi.org/10.1016/S0140-6736(10)60884-7.

[CIT0006] Fritz K., McFarland W., Wyrod R., Chasakara C., Makumbe K., Chirowodza A. (2011). Evaluation of a Peer Network-Based Sexual Risk Reduction Intervention for Men in Beer Halls in Zimbabwe: Results from a Randomized Controlled Trial. AIDS and Behaviour.

[CIT0007] Kalichman S. C. (2010). http://pubs.niaaa.nih.gov/publications/arh333/184-194.htm.

[CIT0008] Kalichman S. C., Simbayi L. C., Kaufmann M., Cain D., Jooste S. (2007). Alcohol use and Sexual Risks for HIV/AIDS in Sub-Saharan Africa: Systematic Review of Empirical Findings. Prevention Science.

[CIT0009] Kalichman S. C., Simbayi L. C., Vermaak R., Cain D., Smith G., Mthembu J. (2008). Randomized Trial of a Community-Based Alcohol-Related HIV Risk-Reduction Intervention for Men and Women in Cape Town South Africa. Annals of Behavioral Medicine.

[CIT0010] Kegeles S. M., Hays R. B., Coates T. J. (1996). The Mpowerment Project: A Community-Level HIV Prevention Intervention for Young Gay Men. American Journal of Public Health.

[CIT0011] Kelly J. A. (2004). Popular Opinion Leaders and HIV Prevention Peer Education: Resolving Discrepant Findings, and Implications for the Development of Effective Community Programmes. AIDS Care.

[CIT0012] Kelly J. A., St Lawrence J. S., Diaz Y. E., Stevenson L. Y., Hauth A. C., Brasfield T. L. (1991). HIV Risk behaviour Reduction Following Intervention with Key Opinion Leaders of Population: An Experimental Analysis. American Journal of Public Health.

[CIT0013] Kelly J., Murphy D., Sikkema K., McAuliffe T., Roffman R., Solomon L. (1997). Randomised, Controlled, Community-Level HIV-Prevention Intervention for Sexual-Risk behavior among Homosexual Men in US Cities. Lancet.

[CIT0014] Ker K., Chinnock P. (2006). Interventions in the Alcohol Server Setting for Preventing Injuries. The Cochrane Database of Systematic Reviews.

[CIT0015] Lewis J. C., Garnett G. P., Mhlanga S., Nyamukapa C., Donnelly C., Gregson S. (2005). Beer Halls as a Focus for HIV Prevention Activities in Rural Zimbabwe. Sexually Transmitted Diseases.

[CIT0016] Mayfield D., McLeod G., Hall P. (1974). The CAGE Questionnaire: Validation of a New Alcoholism Instrument. American Journal of Psychiatry.

[CIT0017] Miller W. R., Rollnick S. (1991). Motivational Interviewing: Preparing People to Change Addictive Behaviour.

[CIT0018] Morojele N. K., Kachieng'a M. A., Nkoko M. A., Moshia K. M., Mokoko E., Parry C. D. H. (2004). Perceived Effects of Alcohol use on Sexual Encounters among Adults in South Africa. African Journal of Drug and Alcohol Studies.

[CIT0019] Morojele N. K., Kachieng'a M. A., Mokoko E., Nkoko M. A., Parry C. D., Nkowane A. M. (2006). Alcohol use and Sexual behaviour among Risky Drinkers and Bar and Shebeen Patrons in Gauteng Province, South Africa. Social Science and Medicine.

[CIT0020] Morojele N., Fritz K., Parry C., Manda O., Nel E. (2007). Development of Reports on Alcohol and HIV Interventions: Recommendations from Formative Research.

[CIT0021] Neuman M. G., Schneider M., Nanau R. M., Parry C., Chersich M., Maddock J. (2012). The Relationship between Alcohol Consumption and Human Immunodeficiency Virus Infection and Risk: A Systematic Literature Review of High-Risk Groups, with a Focus on South Africa. Public Health – Social and Behavioral Health.

[CIT0022] NIMH Collaborative HIV/STD Prevention Trial Group (2010). Results of the NIMH Collaborative HIV/Sexually Transmitted Disease Prevention Trial of a Community Popular Opinion Leader Intervention. Journal of Acquired Immune Deficiency Syndrome.

[CIT0023] Parry C. D. H., Rehm J., Morojele N. K. (2010). Is there a Causal Relationship between Alcohol and HIV: Implications for Policy, Practice and Future Research. African Journal of Drug and Alcohol Studies.

[CIT0024] Peltzer K., Ramlagan S., Gliksman L. (2006). Responsible Alcoholic Beverages Sales and Services Training Intervention in Cape Town: A Pilot Study. Journal of Psychology in Africa.

[CIT0025] Reilly D., Van Beurden E., Mitchell E., Dight R., Scott C., Beard J. (1998). Alcohol Education in Licensed Premises using Brief Intervention Strategies. Addiction.

[CIT0026] Roerecke M., Obot I. S., Patra J., Rehm J. (2008). Volume of Alcohol Consumption, Patterns of Drinking and Burden of Disease in Sub-Saharan Africa. African Journal of Drug & Alcohol Studies.

[CIT0027] Russell M., Martier S. S., Sokol R. J., Mudlar P., Bottoms S., Jacobson S. (1994). Screening for Pregnancy Risk Drinking. Alcoholism: Clinical and Experimental Research.

[CIT0028] Sandøy I. F., Siziya S., Fylkesnes K. (2008). Lost Opportunities in HIV Prevention: Programmes Miss Places where Exposures are Highest. BMC Public Health.

[CIT0029] Shisana O., Rehle T., Simbayi L. C., Zuma K., Jooste S., Pillay-van-Wyk V. (2009). South African National HIV Prevalence, Incidence, Behaviour and Communication Survey 2008: A Turning Tide among Teenagers?.

[CIT0030] Shuper P. A., Neuman M., Kanteres F., Baliunas D., Joharchi N., Rehm J. (2010). Causal Considerations on Alcohol and HIV/AIDS – A Systematic Review. Alcohol and Alcoholism.

[CIT0031] UNAIDS & WHO (2009). AIDS Epidemic Update: December 2009.

[CIT0032] Van Beurden E., Reilly D., Dight R., Mitchell E., Beard J. (2000). Alcohol Brief Intervention in Bars and Taverns: A 12-month follow-up Study of Operation Drinksafe in Australia. Health Promotion International.

[CIT0033] Weir S. S., Pailman C., Mahlalela X., Coetzee N., Meidany F., Boerma J. T. (2003). From People to Places: Focusing AIDS Prevention Efforts where it Matters Most. AIDS.

[CIT0034] Williamson L. M., Hart G. J., Flowers P., Frankis J. S., Der G. J. (2001). The Gay Men's Task Force: The Impact of Peer Education on the Sexual Health behaviour of Homosexual Men in Glasgow. Sexually Transmitted Infection.

[CIT0035] Woolf-King S. E., Maisto S. A. (2011). Alcohol use and High-Risk Sexual behavior in Sub-Saharan Africa: A Narrative Review. Archives of Sexual Behavior.

[CIT0036] World Health Organization (2004). Global Status Report on Alcohol 2004.

